# Retraction of the Research Article: “Individualistic sensitivities and exposure to climate change explain variation in species’ distribution and abundance changes”

**DOI:** 10.1126/sciadv.1600819

**Published:** 2016-04-29

**Authors:** Georgina Palmer, Jane K. Hill, Tom M. Brereton, David R. Brooks, Jason W. Chapman, Richard Fox, Tom H. Oliver, Chris D. Thomas

**Affiliations:** 1 Department of Biology, University of York, Wentworth Way, York YO10 5DD, UK.; 2 Butterfly Conservation, Manor Yard, East Lulworth, Wareham, Dorset BH20 5QP, UK.; 3 AgroEcology Department, Rothamsted Research, Harpenden, Hertfordshire AL5 2JQ, UK.; 4 Environment and Sustainability Institute, University of Exeter, Penryn, Cornwall TR10 9EZ, UK.; 5 NERC (Natural Environment Research Council) Centre for Ecology and Hydrology, Benson Lane, Crowmarsh Gifford, Oxfordshire OX10 8BB, UK.

In our recent Research Article “Individualistic sensitivities and exposure to climate change explain variation in species’ distribution and abundance changes” (*1*), we presented an analysis relating species-specific measures of sensitivity and exposure to climate, to species’ recent population changes. While our measure and interpretation of species’ climate sensitivity remain correct, we now recognize our interpretation of the exposure measure was inaccurate: Our climate models included an intercept, representing a nonzero average population growth rate; thus, the exposure measure incorporated not only climate effects but other nonclimatic—and potentially unmeasured climatic—effects as well. While our results still demonstrate that a significant proportion of variation in population trends can be explained by exposure and sensitivity, the correct interpretation of the exposure measure means that the explained variation is not solely due to climate. As such, our conclusion that a large proportion of variation in population changes can be explained by individualistic responses to climate is misleading. Given this, and to avoid confusion, we are wholly retracting the Research Article, and we apologize that this was not picked up sooner.
